# An analysis of stakeholder networks to support the breastfeeding scale-up environment in Mexico

**DOI:** 10.1017/jns.2020.4

**Published:** 2020-03-09

**Authors:** Gabriela Buccini, Kassandra L. Harding, Isabel Ferré Eguiluz, Cara B. Safon, Amber Hromi-Fielder, Teresita González de Cosío, Rafael Pérez-Escamilla

**Affiliations:** 1 Department of Social and Behavioral Science, Yale School of Public Health, New Haven, CT, USA; 2 Health Department, Universidad Iberoamericana, Mexico City, México; 3 Department of Health Law, Policy and Management, Boston University School of Public Health, Boston, MA, USA

**Keywords:** Breastfeeding, Stakeholder participation, Nutrition policy, Implementation science, BBF, Becoming Breastfeeding Friendly, BMSC, breastmilk substitute companies, ConMéxico, Mexican Council of the Consumer Products Industry, EBF, exclusive breastfeeding, NGO, non-government organisation, PAHO, Pan-American Health Organization, SHCP, Ministry of Finance and Public Credit, SSAF, Federal Ministry of Health

## Abstract

Little information exists on how to garner political commitment to strengthen large-scale breastfeeding policies and programmes by targeting key decision makers. The present study aims to map and describe the influence of stakeholders involved in breastfeeding policy and programming and identify opportunities to strengthen the breastfeeding-friendly environment in Mexico. A total of nine key informants from seventeen stakeholder organisations were selected based on their in-depth knowledge of the breastfeeding environment in Mexico and were individually interviewed using Net-Map methodology. This participatory interview technique combines stakeholder mapping, social network analysis and influence mapping to identify relevant stakeholders. Participants identified a total of fifty-five stakeholders shaping breastfeeding programmes and policies through four domains of influence: commands (*n* 32 stakeholders), dissemination (*n* 40), funding (*n* 35) and technical assistance (*n* 37). The Federal Ministry of Health emerged as the most influential stakeholder of breastfeeding policy and programming decisions in Mexico among all domains of influence. The Ministry of Finance and Public Credit as well as the National Institute of Public Health were identified as additional key stakeholders providing funding and technical assistance to the Federal Ministry of Health, respectively. Engaging identified key stakeholders can generate a multisectoral commitment to breastfeeding and strengthen the breastfeeding-friendly environment in Mexico.

The short- and long-term benefits of optimal breastfeeding practices on infant and maternal health are well documented^(1,2)^. Both the WHO and UNICEF recommend early initiation of breastfeeding, exclusive breastfeeding (EBF) during the first 6 months of life, and continued breastfeeding for at least the first 24 months of age. Yet, in 2018, the global prevalence of EBF was only 41 %^(3,4)^. The 2030 WHO target for optimum breastfeeding practices, including at least 70 % of EBF^(4)^, will not be met unless a supportive policy environment is established to effectively scale up breastfeeding programmes at a national level^(5–8)^. Key actions needed to bolster the supportive policy environment to promote breastfeeding include ensuring (1) increase funding of breastfeeding programmes, including maternity protection in the workplace, (2) compliance with the Baby-Friendly Hospital Initiative, (3) access to breastfeeding counselling and training, (4) availability of community support programmes, (5) continuous monitoring, and (6) regulation of the potential impact of formula companies and associated marketing practices on breastfeeding rates^(4,5,7,9,10)^.

Despite these known evidence-informed actions that provide the minimum conditions needed to support breastfeeding women, many countries have not been able to effectively implement such key actions^(1,4,7,11)^. This may be in part due to a lack of political commitment^(4,12)^. A recent landscape analysis on political commitment for programmes to protect, promote and support breastfeeding found that just one in forty-four high-level stakeholders representing a wide range of actors in the global policy community for breastfeeding (e.g. UNICEF, WHO/Pan-American Health Organization (PAHO), the World Bank, donor agencies, academics, non-government organisations (NGO), civil society organisations and consultants) rated breastfeeding as a high-political priority^(13)^. Stakeholder network analysis has been successfully used to inform explicit strategies to engage diverse stakeholders in the formation and implementation of infant and young child feeding policies in Southeast Asia^(14–19)^. However, there is little information on how to garner political commitment towards implementing appropriate large-scale breastfeeding policies and programmes by systematically targeting key stakeholders and decision makers. This study addresses this gap by reporting on a stakeholder mapping and network analysis conducted among stakeholders in Mexico following an assessment of the country's breastfeeding-enabling environment in 2016^(20)^.

Mexico has one of the lowest EBF rates in Latin America and the Caribbean, and the prevalence has further declined in recent years. Between 2006 and 2012, the prevalence of EBF among 0- to 5-month-old infants decreased from 22·3 to 14·5 %, and only increased slightly to 16 % in 2016^(20)^. This decline, which was especially pronounced in rural areas and among socio-economically vulnerable groups^(20)^, resulted in the development of Mexico's National Strategy for Breastfeeding Action^(21)^. In 2016, a Mexican breastfeeding expert committee assessed the breastfeeding-friendly environment through the application of the Becoming Breastfeeding Friendly (BBF) toolbox^(5,6,20,22)^. The BBF committee of breastfeeding experts in Mexico identified several gaps explaining Mexico's low breastfeeding rates: (1) lack of monitoring and enforcement mechanisms to regulate the WHO Code of Marketing of Breastmilk Substitutes; (2) insufficient maternity leave for working women in the formal sector (12 weeks) and non-existent maternal leave for those working in the informal economy; (3) lack of a specific national budget line to promote, protect and support breastfeeding; (4) health provider training does not meet the minimum breastfeeding and human lactation education curriculum standards recommended by the WHO; (5) only 11 % of the hospitals comply with the Baby-Friendly Hospital Initiative^(20)^. The BBF assessment resulted in the development of recommendations tailored to the country's needs.

To better understand how to maximise the benefit of Mexico's BBF recommendations, this stakeholder analysis as far as we know is the first to investigate the breastfeeding governance system in Mexico by mapping and describing the influence of stakeholders involved in sectors affecting breastfeeding. Identifying and characterising different domains of influence within breastfeeding networks can illuminate pathways to engage stakeholders in future efforts to strengthen the implementation of breastfeeding policies and programmes in Mexico.

## Methods

### Ethical disclosure

This study was conducted according to the guidelines laid down in the Declaration of Helsinki and all procedures involving human subjects were approved by the Ethics Committee from Universidad Iberoamericana. Written informed consent was obtained from all subjects.

### Stakeholder analysis

A stakeholder analysis was carried out using Net-Map, a methodology that has been used to visualise stakeholders' interactions as well as the way these interactions influence the policy decision-making process^(16)^. Net-Map is a participatory interview technique combining stakeholder mapping, social network analysis and power mapping activities. This technique was developed by the International Food and Policy Research Institute (IFPRI) and has been successfully used in the maternal–child nutrition field^(14–19,23,24)^.

The breastfeeding stakeholder analysis included the following three activities to identify and visualise decision makers' influence on the decision making required to translate the 2016 BBF policy recommendations into action within Mexico: (1) stakeholder mapping to identify stakeholders who influence breastfeeding policy and/or programming; (2) social network analysis to allow for the assessment of mechanisms and pathways that connect stakeholders to one another considering four domains of influence: command, dissemination, funding and technical assistance as defined in Table 1; and, (3) power mapping to highlight who holds greater/lesser influence within each domain of influence.

**Table 1.** Key terms and domains of influence*

	Definition
Key term	
Stakeholder	Organisations or individuals with interest in the issues being addressed, whether they are actively or potentially involved in affecting breastfeeding outcomes or passively affecting them An organisation or individual with decision-making power about policy implementation
Influence	Level of influence that an organisation or individual has to make a change in the breastfeeding governance system. The ‘extent of influence’ refers to the power a stakeholder has in translating a policy recommendation into action, i.e. implementing or scaling it up
Breastfeeding governance system	The political decision-making process involved in the translation of policy recommendations into action
Breastfeeding friendly environment	An environment – a country, a state, a workplace, etc. – where breastfeeding is protected, promoted and supported to enable mothers to breastfeed as long as they plan/wish
Domains of influence	
Command	Stakeholders linked by giving or receiving commands (for example, one stakeholder tells the other that it must do something)
Funding	Stakeholders linked by giving or receiving money or financial incentives (for example, one stakeholder funds projects within another)
Technical assistance	Stakeholders linked by giving or receiving technical assistance (for example, one stakeholder offering advice to another)
Dissemination	Stakeholders linked by the dissemination of information (for example, one or both stakeholders spread information about what one or both have developed)

* Adapted from the International Food and Policy Research Institute (IFPRI) policy study^(25)^.

### Identification of study participants

A preliminary list of potential participants to be interviewed was identified based on the list of 275 individual stakeholders who attended the 2016 Mexico BBF Policy Recommendation Dissemination event^(20)^. This list of stakeholders included individuals from government agencies, international organisations, academic organisations as well as civil society represented by NGO (see Table 2 for a full list of stakeholders' organisations). Individual stakeholders from the same organisation were grouped within that organisation by two co-authors (I. F. E. and T. G. de C.) with comprehensive knowledge of breastfeeding policy in Mexico. This process generated a final list of sixteen organisations with potential participants. Then, these two co-authors (I. F. E. and T. G. de C.) ranked the potential participants from 1 to 5 (1 = not at all influential, 5 = most influential), based on how much influence their organisation had relative to other organisations on changing breastfeeding policy or programmes. Once rankings were completed, ten stakeholders within the ten highest ranked organisations were selected to participate in the study. Factors such as job position within the organisations as well as availability of the individual to be interviewed were considered.

**Table 2.** Full list of stakeholders' organisations represented in the 2016 Mexico Becoming Breastfeeding Friendly (BBF) Policy Recommendation Dissemination event

Government agencies
Federal Ministry of Health
Chamber of Deputies
National Center for the Health of Children and Adolescents
National Center for Gender Equity and Reproductive Health
National Commission for Social Protection in Health
Mexican Social Security Institute
National Institute of Public Health Ministry of Labour and Social Security
Undersecretary of Prevention and Promotion of Health
National System for the Protection of Children and Adolescents
International organisations
United Nations International Children's Emergency Fund (UNICEF)
International Baby Food Action Network (IBFAN)
Pan-American Health Organization (PAHO)
Academic organisations
Universidad Iberoamericana
Autonomous Technological Institute of Mexico
Civil society represented by non-government organisations
Food Orientation Center
Un Kilo de Ayuda
Association of Lactation Consultants in Mexico (ACCLAM)

The ten participants identified via the ranking process represented government (*n* 5), NGO (*n* 3), academia (*n* 1) and an international organisation (*n* 1) (Table 3). Participants were sent written invitations, explained the purpose of the stakeholder mapping activity, and asked to participate in a one-on-one, in-person session in a private meeting place. Between November and December 2017, a total of nine participants were interviewed. One interview was cancelled due to difficulties in scheduling an appropriate time for conducting the activity.

**Table 3.** Participant characteristics, breastfeeding social networks in Mexico

Stakeholder group	Number of participants	Organisations represented
Government	5	SIPINNA, CENSIA, INSP, STPS, CNEGSR
Non-government organisations	3	Un Kilo de Ayuda, Lactation Consultant (IBCLC)
Academia	1	ITAM
International organisations	1	UNICEF

SIPINNA, National System for the Protection of Children and Adolescents; CENSIA, National Center of Children and Adolescents' Health; INSP, National Institute of Public Health; STPS, Ministry of Labor and Social Security; CNEGSR, National Center for Gender Equity and Reproductive Health; IBCLC, International Board-Certified Lactation Consultant; ITAM, Autonomous Technological Institute of Mexico; UNICEF, United Nations International Children's Emergency Fund.

### Data collection tool

Definition of key terms and domains of influence used during the interviews, as well as the interview guide, were adapted *a priori* from the International Food and Policy Research Institute (IFPRI) policy study^(25)^ (Table 1). Specifically, the interview guide asked about: (a) stakeholder identification (who the stakeholders involved are); (b) identification of links (how the stakeholders are linked), including specification of four domains of influence (command, funding, technical assistance and dissemination); and (c) power mapping (how influential the stakeholders are). The guide was translated into Spanish by one co- author (I. F. E.).

### Interviews

All staff conducting Net-Map interviews were trained prior to interview administration via an online training session by three co-authors (C. B. S., G. B. and A. H.-F.) with materials adapted from the International Food and Policy Research Institute (IFPRI) Net-Map Toolbox Manual^(25)^.

**Fig. 1.**
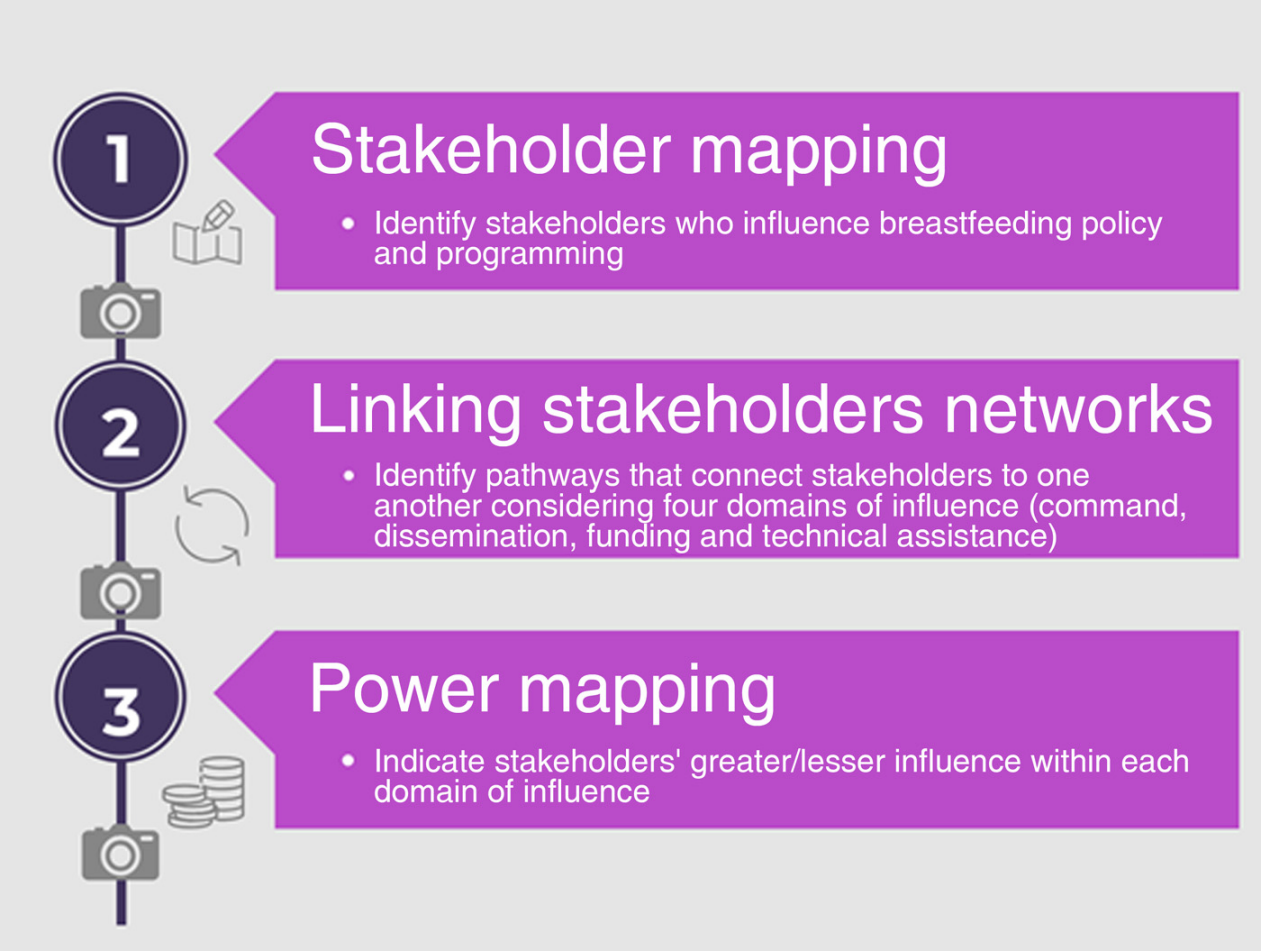
Overview of the stepwise process to apply the Net-Map activity to analyse breastfeeding stakeholders in Mexico. Adapted from Schiffer & Waale^(23)^.

Each participant engaged in an interactive discussion guided by a co-author using the interview guide. A physical mapping of the data based on each interview was generated. An overview of the Net-Map activity is shown in Fig. 1. First, stakeholder mapping was conducted by asking the participant to identify the stakeholders who were most influential, i.e. who was playing an important role in breastfeeding policy decision making. Then, participants classified stakeholders into four stakeholder groups: Government, NGO, Academic or Other. Second, participants were asked to link stakeholder networks by using arrows demonstrating the flow of influence from one organisation to another. Third, for power mapping, participants were asked to rank each stakeholder's organisations on a scale of 0 (does not at all influence the formulation of breastfeeding policy and programmes) to 5 (influences the formulation of breastfeeding policy and programmes to the highest degree) to determine the extent to which each organisation had relative influence on policy and programming decision making. All steps comprising the Net-Map activity were documented using photographs and audio recordings. Nine Net-Map interviews were conducted and each lasted 60 to 90 min.

### Data management

Data from each interview were entered into a separate Microsoft Excel sheet, representing stakeholder characteristics (i.e. stakeholder names and stakeholder group allocation) and links among stakeholders, such that each connection between stakeholders had a ‘source’ and ‘target’. Stakeholder characteristics were compared across interviews to confirm consistency of stakeholder names and stakeholder groups, any inconsistency was recoded based on the majority of responses across interviews. Afterwards, two stakeholder groups were added: International organisations and Demand side/Civil society. Discrepancies in coding were resolved via a consensus process between two co-authors (G. B. and I. F. E.).

Data for stakeholder links were appended in Microsoft Excel. Then, data were imported into Gephi 0.9.2 to generate directed network maps using the Yifan Hu algorithm and social network descriptive statistics as defined in Table 3.

### Data analysis

First, a distribution of stakeholders was reported across the four domains of influence, including a description of stakeholder identification and group classification as well as the relative influence of stakeholders with respect to shaping the breastfeeding environment in Mexico. Stakeholder relative influence was weighted by number of citations across the nine interviews, generating an average weighted influence with a potential range of 0 to 5. To streamline the analysis, stakeholders who were cited in only one interview and had a weighted relative influence equal to 0 were dropped from the analysis (*n* 12) because they were unlikely to serve as key influencers of the breastfeeding political decision-making process in Mexico. Then, the size of the network including number of nodes, links and unique links was described.

Second, a map was generated for each domain of influence. A stakeholder was indicated by a node in the network and connected to one another by links represented by lines and arrows. Nodes were colour-coded by stakeholder groups and sized proportionate to the level of weighted relative influence. Larger relative size corresponded to higher relative influence^(14,15,18)^. Stakeholders in the network and links are reported as stated by participants. Hence, the maps are representative of the views and experiences of participants. Social network analysis measures of cohesion (density and distance) and measures of centrality (in-degree, out-degree, and betweenness as well as mean degrees), as defined in Table 4, were used to describe each network.

**Table 4.** Key social network terms and statistics*

Statistics	Shows	Explanation
Size reflects the distribution of stakeholder groups		
Number of nodes	Size of the network	Number of stakeholders in the network
Number of links	How ‘busy’ the network is in total	Number of connections between stakeholders in the network (in total)
Number of unique links	How ‘busy’ the network is, eliminating relationships that are duplicated	Number of connections between individuals in the network, with duplicates removed
Cohesion reflects the interconnectedness of stakeholders in a network		
Distance	Proximity of nodes to one another	Average number of links between nodes. Where distances are great, it may take a long time for information to diffuse across a population; moreover, stakeholders who are closer to more others may be able to exert more power than those who are more distant
Density	The extent to which nodes are interconnected	The proportion of all links that are actually present out of all possible links. Density is a ratio that can range from 0 to 1; the closer to 1 the density is, the more interconnected the network is
Centrality reflects the prominent stakeholders within a network		
Mean degrees	How central (on average) nodes in the network are	Average number of links that pass through the nodes
In-degree	Quantifies the inputs, or directions, received by a stakeholder from the other stakeholders in the network	Measures the number of links directed at a stakeholder, representing the received input from a particular network
Out-degree	Quantifies the links of a stakeholder provided to other stakeholders in the network	Measures the number of links from a stakeholder directed to other stakeholders in the network, representing the input provided to a particular network
Betweenness	Represents the control a stakeholder has over the flow of inputs across a network	Measures the number of times a stakeholder connects subgroups within a network

* Adapted from Home Office (2016)^(33)^; Hawe *et al*. (2004)^(32)^.

## Results

### Distribution of stakeholders involved in the breastfeeding governance system in Mexico

#### Stakeholder identification

**Fig. 2.**
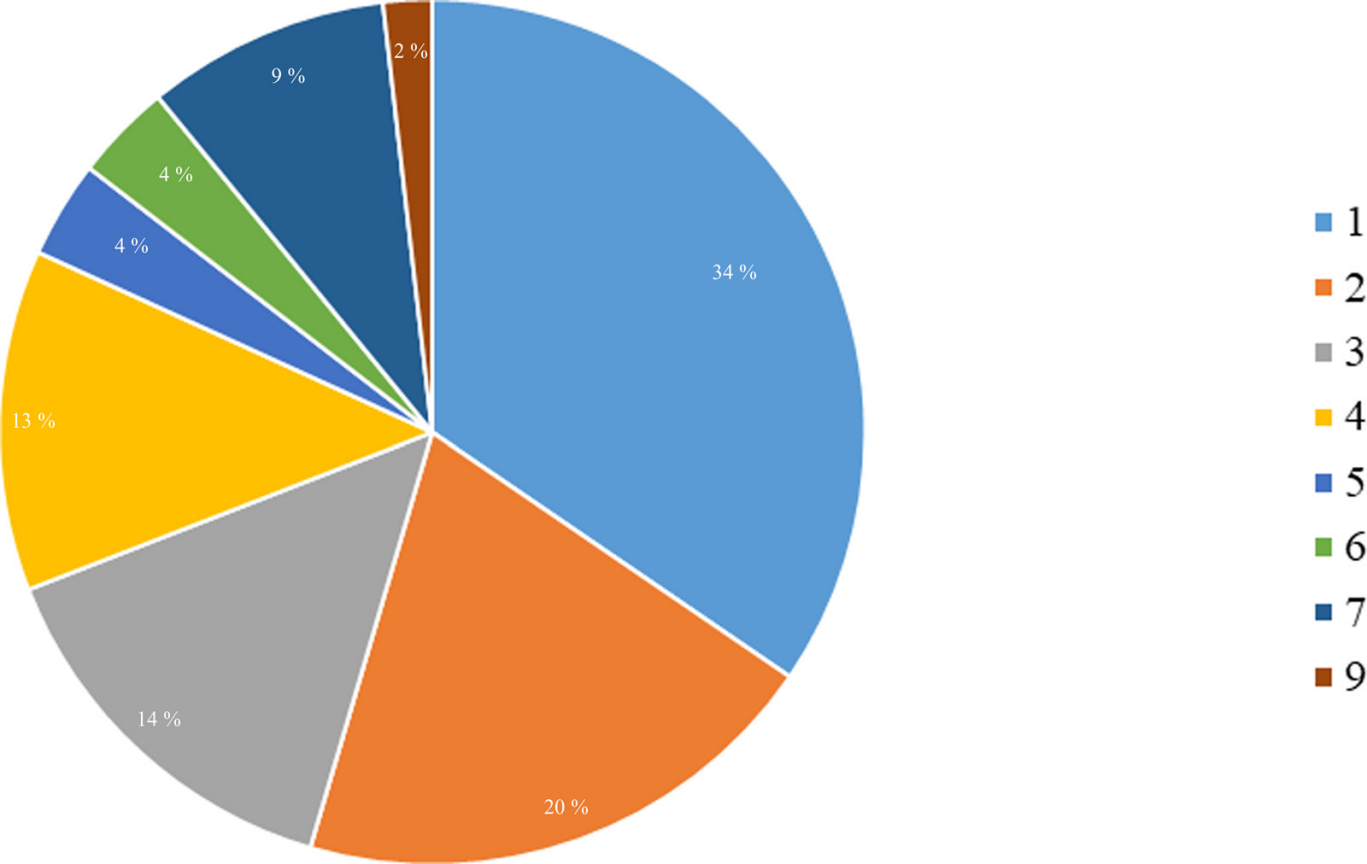
Percentage of breastfeeding stakeholders in Mexico representing the number of citations across the nine interviews. Numbers reflect the number of times a stakeholder was cited across the nine interviews, ranging from 1 to 9.

A total of fifty-five stakeholders were identified across the nine interviews (Fig. 2). Approximately 30 % of stakeholders (*n* 16) were cited within four to seven interviews, and only one stakeholder (Ministry of Labour and Social Security (STPS for its acronym in Spanish)) was cited in all nine interviews.

#### Stakeholder group classification

Almost half of the identified stakeholders were from the Government sector (*n* 27), followed by NGO (*n* 8), International organisations (*n* 7), Other (*n* 7), Academia (*n* 5), and lastly Civil society/Demand side (*n* 1). Other groups included breastmilk substitute companies (BMSC), milk producers (Productores de Leche), Mexican Council of the Consumer Products Industry (ConMéxico), professional associations representing paediatricians (AOP), lactation consultants (Association of Lactation Consultants in Mexico (ACCLAM)), hospitals and work centres (CT).

#### Stakeholder relative influence

**Fig. 3.**
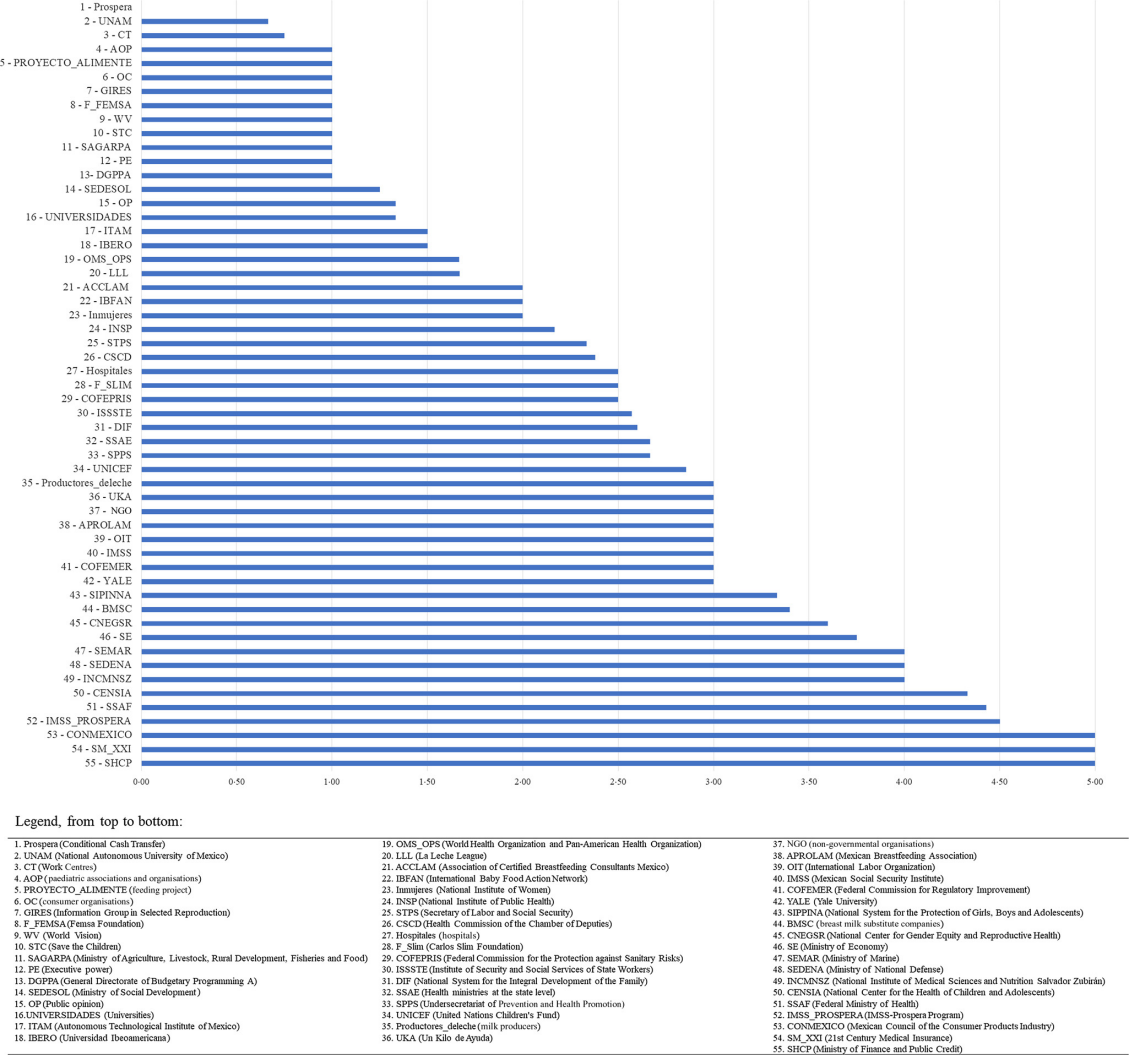
Average weighted influence for all breastfeeding stakeholders in Mexico; ranked lowest to highest.

Stakeholders were ranked by their relative influence with respect to shaping the breastfeeding environment in Mexico (Fig. 3). 21st Century Medical Insurance (SM_XXI), the Ministry of Finance and Public Credit (SHCP), the Mexican Council of the Consumer Products Industry (ConMéxico), Prospera's Mexican Social Security Institute (IMSS/Prospera), the Federal Ministry of Health (SSAF) and the National Center of Children and Adolescents' Health (CENSIA) were the top six stakeholders that had the greatest relative influence (i.e. between 4 and 5 out of 5). Interestingly, all but one (ConMéxico) of these stakeholders represented the government sector.

#### Size of network

There were 444 links identified in the nine interviews, with 319 unique links connecting the fifty-five stakeholders identified. The technical assistance network had the most unique number of links identified (*n* 113), followed by dissemination (*n* 79), command (*n* 71) and funding (*n* 56).

### Networks of breastfeeding stakeholders in Mexico

**Fig. 4.**
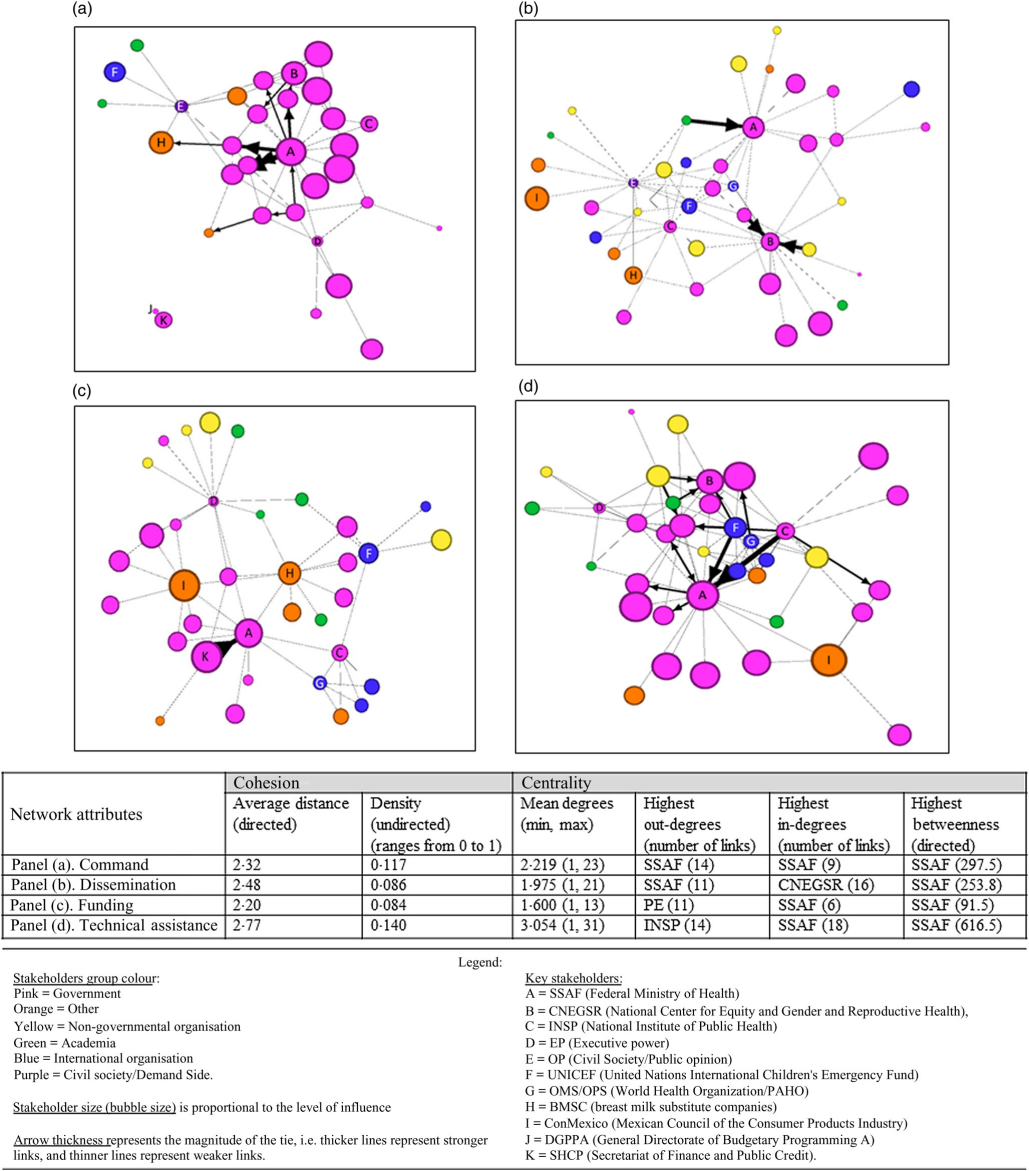
Maps of Mexico's breastfeeding policy stakeholders sized by sum reported influence, colour-coded by stakeholder groups and stratified by domains of influence: commands links (a), dissemination links (b), funding links (c) and technical assistance (d).

Network maps by each of the four domains of influence are described below (Fig. 4(a)–(d)).

#### Command network

A total of thirty-two stakeholders were connected by seventy-one unique command links that were mostly comprised of government stakeholders with a smaller presence of stakeholders from International Organisations, Civil society/Demand side and other sectors (Fig. 4(a)).

For the command network, a centralised power on government stakeholders was observed: the SSAF (i.e. the Federal Ministry of Health) received the highest value for all the centrality network measures, indicating that the SSAF was the most influential over the flow of command between stakeholders by its position in the network (betweenness centrality = 297·5). Additionally, the SSAF received a high level of commands (in-degree, twenty-eight links) from other government organisations and provided commands (out-degree, twenty-six links) to other government organisations. Even though Academia, International organisations, Other and Civil society/Demand side (OP) stakeholders were present in this network, they showed a relatively low level of influence.

Furthermore, a lack of cohesion among stakeholders within this network was observed. For instance, two stakeholders were not connected with the full network; this break in linkage can have an impact on the ability of commands to reach these stakeholders or for the network to receive commands from these stakeholders. Interestingly, both stakeholders, the General Directorate of Budgetary Programming A (DGPPA) and the SHCP, direct the budget regarding breastfeeding programmes.

#### Dissemination network

Within the network for dissemination, forty stakeholders were connected by seventy-nine unique links with multisectoral stakeholders’ participation across stakeholder groups. (Fig. 4(b)).

For the dissemination network, the SSAF had a centralised position in the network and provided dissemination to the largest number of other stakeholders (out-degree, eleven links), indicating the high extent of its influence with respect to the flow of dissemination between stakeholders who were mostly government stakeholders. The National Center for Equity and Gender and Reproductive Health (CNEGSR) received the highest level of dissemination (in-degree, sixteen links).

Despite the heavy presence of key government stakeholders in disseminating breastfeeding information, a central position of International Organisations (UNICEF and WHO/PAHO) was identified. Additionally, a cluster of stakeholders formed by government, NGO, Civil society/Demand side (OP) and organisations representing the Other group also had central positions.

#### Funding network

The network representing funding links included thirty-five stakeholders connected by fifty-six unique links. This network included multisectoral stakeholder participation with a large presence of stakeholders from the government sector (Fig. 4(c)).

The SSAF was strongly linked and targeting the SHCP, which oversees the allocation of funds across government sectors. Also, the SSAF received funding support from the greatest number of stakeholders (in-degree, eleven links) including Government, International organisations and other stakeholder groups (BMSC and ConMéxico). The Executive Power (PE) (i.e. the organisation responsible for executing and enforcing the law as well as the budget) provided direct funding support to the greatest number of stakeholders (out-degree, sixteen links) including Government, Academia and NGO.

While the funding network was consistent with other networks presented in this analysis in that the government was highly influential based on measures of centrality and network positioning, a weak cohesion (density and distance) among stakeholders in this network was observed. Differently from the other social networks of breastfeeding in this analysis, conglomerates of stakeholders forming small clusters linked by key stakeholders were identified. The stakeholders linking these clusters were classified as the ‘Other’ group (ConMéxico and BMSC); both are related to the industry of breastmilk substitutes and targeted government, international organisations (UNICEF and WHO/PAHO) and academic stakeholders.

#### Technical assistance

A total of thirty-seven stakeholders were connected by 113 unique links via technical assistance. This network is heavily consolidated around government stakeholders who also have more influence (stakeholder size). A smaller presence of stakeholders from the Other group, NGO and Academia was identified (Fig. 4(d)).

The SSAF had a centralised position within the network, meaning it exerts high influence over the flow of technical assistance and received technical assistance from the greatest number of stakeholders (in-degree, eighteen links), mostly from the government sector but also from international organisations (UNICEF and WHO/PAHO) and academic organisations within Mexico. The National Institute of Public Health (INSP) provided technical assistance to the greatest number of stakeholders in the network (out-degree, fourteen links), mostly to government stakeholders, but also to international organisations. Among international organisations, UNICEF played a key role in providing technical assistance to different stakeholder groups. Nevertheless, although greater cohesion measures were observed within this network compared with other breastfeeding networks in this analysis, the distance among stakeholders observed was still far from each other.

## Discussion

To our knowledge, this stakeholder analysis is the first to investigate the breastfeeding governance system in Mexico. The stakeholder analysis described four different domains of breastfeeding networks heavily led by stakeholders from the government sector. As expected, the Federal Ministry of Health (SSAF) held the most influential position across all networks, which means that setting the policy agenda for breastfeeding and translating a policy recommendation into action must include efforts to engage the SSAF as primary source or target depending on the specific network domain. Our analysis also identified a multisectoral group of stakeholders' organisations shaping the breastfeeding networks which demonstrates the importance of breastfeeding in the agenda of different entities. Understanding these linkages can inform explicit strategies to engage such key stakeholders^(18)^. However, the limited linkages among stakeholders' organisations involved in budgeting and the lack of civil society engagement in the breastfeeding networks were identified as potentially major obstacles to advance in the effective formulation and implementation of breastfeeding policies and programming in Mexico^(6,7,20)^.

Our findings indicate a lack of cohesion across stakeholders in each breastfeeding network, which can have an impact on the breastfeeding policy and programming in different ways. For instance, in the command network, the centralised influence of government stakeholders (primarily SSAF) might make an impact on the speed at which the command diffuses among additional stakeholders within the network; in the funding network, the weak cohesion of the network might have an impact on funding support, mechanisms of funding and definition of competing priorities. On the other hand, the technical assistance network presented greater cohesion measures compared with other breastfeeding networks, which potentially indicate that technical assistance messages will reach each stakeholder in the network, although the speed might not be as desired due to the great distance between stakeholders.

These findings can be understood as either a threat or an opportunity to creating a strong enabling environment for breastfeeding in Mexico. The situation can be perceived as a threat when considering the speed at which the commands and information are diffused among stakeholders as well as in the definition of priorities of technical assistance and funding. For example, the lack of cohesion within a network has been shown to be associated with competing priorities among stakeholders as well as generating higher costs to the policy-making process^(26)^. This may be due to the need for increased multiple efforts to influence or reach a target stakeholder towards a common goal, in the case of this analysis to enable a breastfeeding-friendly environment. On the other hand, this finding could be viewed as an opportunity to further enable the breastfeeding-friendly environment in Mexico when enhancing the multisectoral commitment for breastfeeding. Moreover, our findings documented that in Mexico there is room to build more ties and partnerships; specifically, civil society, NGO, international organisations and academia have the potential to be more integrated and have a more influential position across the networks^(27)^. Evidence has shown that the governance system to create the enabling environment for breastfeeding should encompass complex multisectoral interactions between public and private entities, including a strong presence of civil society (advocacy) with the aim of reaching shared goals and actions to improve breastfeeding^(4,27)^.

In this sense, any process to assess the breastfeeding environment like BBF does^(5,6,22)^ can help intensify the interaction of multisectoral stakeholders^(27)^ towards an informed, evidence-based consensus about the needs of the country regarding the scale up of breastfeeding programmes to ultimately improve optimal breastfeeding practices^(6)^. In the 2016 BBF assessment in Mexico, the lack of funding for breastfeeding represented a major issue identified by breastfeeding experts^(20)^. Indeed, the present analysis identified stakeholders with high influence and potential competing priorities, including conflicts of interest. This could signify a lack of a shared policy agenda and goals^(28)^. For example, in the funding network, stakeholders within the industry of breastmilk substitutes (BMSC and ConMéxico) were identified as powerful stakeholders influencing the funding network (against breastfeeding interests) by creating a conflict of interest through provision of funds to government stakeholders. Furthermore, in the command network, government stakeholders involved in budgeting (General Directorate of Budgetary Programming A (DGPPA) and SHCP) were not connected with the full network. This suggests that there is limited communication among key stakeholders which may be making an impact on the lack of funding available for the scaling up of breastfeeding actions as identified by breastfeeding experts in the 2016 BBF assessment^(20)^. Thus, our stakeholder mapping identified different aspects of how to strengthen the breastfeeding network within Mexico, including the importance of building a multisectoral network and the importance of defining a common goal for breastfeeding within each network^(7,28)^.

This study has some limitations and strengths. First, our selection of only one participant per organisation to report the social networks for breastfeeding could have limited the quality of such reports^(29)^; perhaps including more than one participant per organisation would have strengthened results by including broader perspectives. On the other hand, we selected participants from different organisations, which allowed us to capture a comprehensive perception of each breastfeeding network due to the fact that participants were familiar with different aspects of each breastfeeding network^(30)^. In this sense, individual mapping interviews have been conducted in several Net-Map studies showing the reliability and validity of the data collected^(30,31)^. We also developed a systematic process to weigh and combine each map (explained in the Methods/analysis section), resulting in, to our knowledge, the first meaningful maps for each breastfeeding network in Mexico (i.e. commands, dissemination, funding and technical assistance). Another strength of our study is the systematic preparation of data collection and training of interviewers^(29,32)^.

In summary, we found that the SSAF is the primary key stakeholder in the policy-making process for breastfeeding in Mexico; thus any changes or call to set up the breastfeeding agenda in Mexico should engage this stakeholder. The limited linkages among stakeholders involved in budgeting and other breastfeeding stakeholders are a major obstacle in the breastfeeding environment in Mexico. Finally, enhancing multisectoral commitment, including strengthening the power/influence of civil society, for breastfeeding is an opportunity to further enable the breastfeeding-friendly environment in Mexico.
